# The High Cost of American Health Care: Understanding the Deeper Roots of the Crisis

**DOI:** 10.1007/s10728-025-00554-x

**Published:** 2025-12-15

**Authors:** Cyril F. Chang, David M. Mirvis, Asos Mahmood

**Affiliations:** 1 Professor Emeritus of Economics, University of Memphis, Memphis, TN USA; 2 Professor Emeritus of Medicine, University of Tennessee Health Science Center, Memphis, TN USA; 3 Center for Health System Improvement, College of Medicine, University of Tennessee Health Science Center, Memphis, TN USA; 4 Department of Medicine-General Internal Medicine, College of Medicine, University of Tennessee Health Science Center, 956 Court Ave Avenue, Memphis, TN 38103 USA; 5 Tennessee Population Health Consortium, University of Tennessee Health Science Center, Memphis, TN USA

**Keywords:** Health care costs, Price transparency, Fee-for-service, Value-based payments, Baumol effect

## Abstract

The recent tragic death of UnitedHealthcare CEO Brian Thompson has renewed public scrutiny of the US health care system and reignited debate over why Americans face disproportionately high health care costs. This essay examines both the widely recognized drivers of excessive costs and the deeper systemic issues that often remain unaddressed yet continue to sustain the crisis. It highlights two critical challenges: the need to target structural barriers and misaligned economic incentives in order to design sustainable reforms, and the persistent political polarization that impedes long-term, meaningful change. The paper concludes by outlining short-term strategies that can generate immediate improvements while laying the foundation for comprehensive and enduring reform.

## Introduction

For decades, the United States (US) has wrestled with the challenge of persistently high health care costs—by far the highest among developed nations, both in absolute terms and as a share of gross domestic product (GDP) [1, 2]. The drivers are well known; expensive medical technologies, high prices for drugs and services, administrative complexity, fee-for-service incentives, and a population burdened by chronic disease [3–5]. Behind these visible factors lie deeper structural forces, such as the political power of entrenched industry stakeholders, fragmented financing arrangements, and the cultural preference for individual choice over collective cost control, that have drawn less attention [6, 7].

Efforts to reform the American health care system have been as protracted and complex as the persistent history of health care inflation. Despite repeated commissions, blue-ribbon panels, and bipartisan task forces, however, these realities have rarely been translated into durable policy change [8, 9]. Policymakers have tended to focus on short-term fixes or politically palatable adjustments, leaving structural issues and misaligned incentives largely untouched. Efforts to address the most conspicuous symptoms (e.g., high prescription drug prices, surprise medical bills, and budget overruns) often sidestep the less visible systemic issues, such as excess utilization of low-value services, market consolidation, and misaligned payment models. The result has been a cycle of recognition without resolution: a wealth of insight into why costs are high, but a persistent inability to convert that understanding into actions capable of bending the cost curve in a lasting way [10, 11].

## The Crisis Point

The tragic shooting and death of UnitedHealthcare CEO Brian Thompson in December 2024 has once again placed the US health care system under intense scrutiny [12]. reigniting debates on why Americans pay so much more for health care than citizens of other developed nations. While the immediate causes of this issue are often well-documented [10, 11, 13]. Understanding the deeper systemic reasons is critical to formulating effective solutions.

Ken Alltucker’s *USA Today* article, published on December 15, 2024, presented seven well-known and “obvious reasons” behind America’s astronomical health care costs [12]. These include a lack of price limits, fee-for-service structures, inflated specialist salaries, administrative costs, opaque pricing, exorbitant and discretionary drug prices, and private equity’s increasing influence. Experts interviewed by Alltucker, like Harvard’s David Cutler, Michael Chernew, and Rice University’s Vivian Ho, have voiced their views on how these and other factors directly impact the system [14, 15]. However, as valid as these points are, they represent only the visible surface of the problem.

In contrast to the current popular views, beneath the “obvious reasons” for excessive health care expenditures lie critical structural undercurrents that have not been widely recognized and openly debated [7, 16]. These deeper issues create conditions that allow the inflationary factors identified by Alltucker and others to thrive. Without addressing these roots, any attempt at reform will be like treating symptoms while ignoring the underlying disease.

This essay thus contributes to the health policy literature by first exploring the well-known factors contributing to these costs. It then thematically delves into the underlying systemic issues that often go unnoticed but continue to fuel the crisis and thwart meaningful reforms. Finally, it addresses two critical challenges: (1) the need to focus reform efforts on structural barriers and economic incentives to develop practical, sustainable solutions, and (2) the difficulty of bridging the political divide that hampers meaningful, long-lasting health care reform.

## The Well-Known Factors of High Health Care Costs

Ken Alltucker’s analysis highlights the most visible reasons for America’s high health care spending. Each of these factors contributes significantly to the issue, but they are often interconnected, creating a system where inefficiencies and cost escalation become inevitable. A clear understanding of these easy-to-see reasons is a necessary step in the discovery of the root causes of the America’ high health care costs. In brief, these are:

- In most countries, governments negotiate or impose price caps on medical services, prescription drugs, and procedures. In the US, however, there are no such *price limits*, allowing providers to charge vastly different rates for the same services. For example, a magnetic resonance imaging (MRI) might cost $400 at one hospital but $4,000 at another [1]. Without clear pricing regulations, consumers are left at the mercy of hospitals, insurers, and pharmaceutical companies [2].
- US hospitals and doctors operate on a *fee-for-service* basis, meaning they are paid for every procedure, test, or treatment performed—regardless of its outcome [4, 11]. This structure creates financial incentives to provide more services rather than focus on improving patient outcomes. As a result, unnecessary treatments and procedures inflate costs while often failing to improve health [17].
- Compared to primary care physicians, *American specialists* are paid significantly more. Specialists also exert considerable influence to protect their interests, which contributes to disparities in care delivery [3]. The American system rewards specialization while underfunding general and primary care, creating an imbalance that drives up costs and limits access to preventive care [15].
- The US health care system is *administratively complex*, with private insurers, Medicare, Medicaid, and employer-based plans creating significant bureaucracy [14]. Administrative expenses account for nearly 25% of health care spending, far more than in other countries [5]. Each layer of paperwork, claims processing, and billing adds to the overall cost.
- Health care pricing remains notoriously opaque [18]. Patients rarely know the cost of services or treatments until after they have received care, leaving them unable to make informed decisions [19]. This *lack of transparency* prevents competition and accountability, allowing providers to charge excessive rates without justification [10, 20].
- Americans pay significantly *higher prescription drugs* than people in other wealthy nations. The pharmaceutical industry’s pricing power is largely unchecked, and the US government does not negotiate drug prices the way other countries do [21]. For example, insulin costs in the US are several times higher than in Canada or Europe [22].
- The growth of *private equity ownership* in health care has led to profit-driven decision-making and behaviors throughout the long and winding health services supply chain [23]. Private equity firms prioritize short-term financial returns over patient care, often increasing costs to payers and patients through aggressive billing practices and cost-cutting measures that harm quality [24, 25].

While these seven factors provide a sharp picture of why health care costs are so high in the US, they do not tell the full story. To address these issues effectively, we must look deeper into the economic and structural dynamics that allow these inflationary trends to persist. Like in the practice of medicine, treating the symptoms alone is often necessary but not sufficient.

## Deeper Systemic Causes of High Costs

Systemic causes of high health care costs are the deeper, underlying or root causes that affect an entire health system rather than a single or a cluster of the parts. Some of these system-wide causes are in plain sight for everyone to see but others are subtle and frequently hidden that often elude our attention. The following are the deeper systemic factors that drive health care costs from, respectively, the supply side and demand side of the health system.

## Supply-Side Factors

### The Baumol Effect

Named after economist William Baumol, this economic concept explains why wages and prices rise in sectors like health care and education even when productivity does not increase [16]. Health care is a labor-intensive industry where human expertise cannot be easily replaced by technology. For example, a surgeon’s skill remains critical regardless of technological advancements. As wages rise across the economy due to productivity growth in other sectors (like manufacturing), health care providers must also increase wages to attract talent, even though productivity in health care cannot match that of other industries. This dynamic causes health care costs to rise steadily [26]. However, the Baumol effect alone cannot justify the extent of price inflation seen in the US. Rather, it interacts with other structural issues to exacerbate the problem. For instance, a lack of competition allows providers to pass these rising costs directly to insurers and patients without resistance. Further, the rapid advancement and growing adoption of artificial intelligence (AI) applications may introduce additional uncertainty in predicting future health care costs.

### A Health Care System that Emphasizes Treatments Over Health

The American health care system is often described as reactive, focusing on treatment rather than promoting health and preventing illness [4]. It delivers advanced, high-quality care to those with financial resources or comprehensive insurance, yet millions face barriers to accessing basic services.

Further, chronic diseases like diabetes and heart disease, which account for 90% of the nation’s health care spending, are often preventable through lifestyle changes and early interventions [27]. Despite the high needs, the US allocates only a small fraction of its health care budget to public health and preventive services when compared to other OECD countries [28]. This imbalance exacerbates health disparities, particularly among low-income and minority communities, where social determinants of health—such as housing, education, income, and access to nutritious food—remain unaddressed [29].

To prioritize individual and population health, the entire system of health care organizations and delivery must shift toward prevention, equitable access, and community-based care, recognizing that health outcomes are shaped more by contextual and individual “social determinants of health,” such as environment, and behavior, than clinical interventions [30].

### Monopolistic Control and Reduced Competition

Perhaps the most significant underlying issue is the lack of competition in the supply of health care services. Like in much of the world, health care markets in the US are often monopolized or dominated by a small number of providers, insurers, and pharmaceutical companies [6, 31]. Unlike markets for most consumer goods, this limited competition enables health care organizations to set prices with little risk of losing business.

The problem is particularly pronounced in the labor market for medical professionals [32, 33]. Entry into the medical profession is tightly controlled through licensing requirements, medical school quotas, and barriers to international medical graduates. These restrictions limit the supply of physicians, driving up wages and creating shortages of care providers.

Additionally, restrictions on labor substitution—such as limits on nurse practitioners’ ability to perform certain tasks—prevent the health care system from operating efficiently [34–36]. Increasing competition by loosening these restrictions and allowing alternative providers to enter the market could reduce costs and improve access to care [37].

### Excessive Government Regulations and Mandates

While regulation is necessary to ensure safety and quality, excessive and burdensome regulations have inflated health care costs in the US. Providers face complex compliance requirements, including billing codes, reporting mandates, and quality measures [7]. These regulations create administrative burdens, including documentation, eligibility verification, and compliance tracking, that require significant resources to navigate, contributing to the high administrative costs mentioned earlier [38] and reduce efficiency [37].

For example, hospitals and physicians must comply with the Health Insurance Portability and Accountability Act (HIPAA), Medicare billing requirements, and state-specific regulations such as the Certificate-of-Need (CON) laws that restrict the construction of new health care facilities and licensure rules that prevent sensible labor substitution. Each layer of regulation increases the cost of doing business, which is ultimately passed on to patients. Streamlining regulations while maintaining safety and quality of care could help reduce costs and improve care delivery [37]. Additionally, simplifying administrative processes can improve efficiency and allow health care providers to focus more on patient care. This balance is crucial for creating a more sustainable and affordable health care system.

## Demand-Side Factors

### The Demand for Medical Technology and Third-Party Payment Systems

The rapid development of medical technology—such as advanced imaging machines, robotic surgeries, and new pharmaceuticals—has revolutionized health care, but it has also driven costs upward. Unlike other countries, the US system encourages widespread and rapid adoption of expensive technologies through third-party payments regardless of whether they improve outcomes [39]. Members of this decentralized system, which include private insurers and public programs such as Medicare and Medicaid make their own decisions without coordinating with each other.

In countries with single-payer systems or price controls, new technologies must demonstrate cost-effectiveness before being adopted widely [7]. In the US, however, there are fewer restrictions on the use of costly innovations. Hospitals often invest in the latest technologies to remain competitive, but this comes at a significant financial cost that is ultimately passed on to patients and employers [4].

### Favorable Tax Treatment of Health Care Benefits

Employer-sponsored health insurance is heavily subsidized through the US tax system. Employers can deduct health care costs, and employee health benefits are not taxed as income. While this policy has made health care more accessible for many Americans, it has also distorted incentives [40]. Tax-free health benefits encourage overutilization of health care services, as individuals perceive these benefits as “free” or low-cost [41]. This drives up demand for low-value services, leading to higher prices across the system [42].

Reforming the tax treatment of health care benefits could encourage more cost-conscious decision-making among both employers and employees. However, this approach is politically challenging, as it involves altering a longstanding and popular system of benefits.

### Health and Health Care Inequities

The US health care system is plagued by profound inequalities, with access to care often dictated by socioeconomic status, race, ethnicity, and geographic location. Low-income, minority and rural populations face higher rates of chronic illnesses, such as diabetes, hypertension, and other cardiovascular diseases, alongside reduced life expectancy due to limited access to quality health care and preventive services [43].

These disparities are further exacerbated by systemic challenges, including the high cost of medical care, underinsurance, and a lack of health care facilities in rural and underserved areas [44]. Structural barriers, such as discrimination and unequal treatment within the health care system, contribute to mistrust and worsened outcomes for marginalized groups [45]. Addressing these inequities requires comprehensive reforms, including expanding Medicaid, ensuring universal insurance coverage, investing in community health programs, and addressing adverse social determinants of health through improving housing, education, income and food security. Indeed, achieving health equity is essential for a just and effective health care system.

## The Elephant in the Room and the Political Environment of Reform

Health care reform in the US is both necessary and achievable, but the nation’s deep political divide significantly hinders progress [8, 46]. The US spends more on health care than any other high-income country but lags in most outcome metrics including life expectancy and chronic disease prevention [47]. Comprehensive reforms—such as expanding universal coverage, replacing the wasteful decentralized health care financing system with a single-payer system, controlling costs, and addressing adverse social determinants of health—are achievable. However, achieving these goals requires bipartisan cooperation and working across the aisle, which remains elusive in today’s polarized political climate.

Partisan disagreements often center around the role of government in health care. For example, Democrats largely support expanding public programs like Medicaid and Medicare, while Republicans favor market-based solutions, such as health savings accounts and deregulation [48]. This divide leads to legislative gridlock, where reforms are either watered down or fail to pass entirely. The Affordable Care Act (ACA) is a prime example; though it expanded coverage to millions, it faced years of legal challenges and attempts at repeal [9].

To overcome these obstacles, policymakers must prioritize collaboration and focus on shared goals, such as reducing costs and improving access [49]. Without bridging the political divide, the US risks perpetuating a health care system that remains inequitable, inefficient, and unsustainable.

## Addressing the Roots: Towards Effective Reform

While overhauling the US health care system will require national consensus and sustained effort, there are several practical reforms that could be implemented relatively quickly and yield meaningful results. These low hanging fruits include targeted reforms that deliver immediate benefits while laying the foundation for long-term change.

A promising first step is to integrate proven and increasingly effective generative *artificial intelligence (AI), telemedicine, and other digital health tools* into care delivery. With the right incentives and guidance, AI can streamline diagnostics, optimize workflows, and handle administrative tasks—freeing up clinicians to focus on patients while reducing costs and improving access [50, 51].

Building on technology-driven gains, another quick win is to *strengthen and modernize hospital price transparency rules*—continuing on the Trump administration’s 2019 initiative but addressing its early shortcomings that remained uncorrected by the Biden administration. Enforcement should have meaningful penalties to ensure compliance, and posted prices must be standardized, searchable, and easy for both patients and employers to use. In addition, transparency works best when paired with incentives, such as cost-sharing designs or rewards, that give consumers a reason to select lower-cost, high-quality care. By focusing first on high-cost, high-variation services—like imaging, surgery, and specialty drugs—policymakers can maximize impact while making the market more competitive and navigable [52].

Policymakers can also *expand proven value-based payment models* such as accountable care organizations, bundled payments, and patient centered medical homes with the help of health AI technologies and tools [50, 53]. These value-based programs have shown they can improve quality and reduce unnecessary treatments by aligning incentives with better patient outcomes rather than sheer service volume [54, 55]. Integrating health AI applications can significantly enhance the efficiency and effectiveness of these payment models while reducing the likelihood of partisan conflict and political entanglement. By focusing on AI-enabled care, transparent pricing, and targeted payment reforms, the nation’s health system can take decisive steps toward cost control—delivering real improvements today while setting the stage for deeper systemic transformation tomorrow.

## Limitations

This essay has a few limitations. It does not provide an exhaustive examination of all drivers of high health care costs, nor does it constitute a systematic review or meta-analyses. Instead, its purpose is to highlight less visible root causes of high costs and the challenges of achieving durable health care reform within the current political climate. The quick fixes suggested here are illustrative rather than prescriptive, intended to show what may be possible in advancing reform while generating meaningful benefits. This paper is not a detailed policy blueprint, and it does not present a comprehensive reform plan. Rather, it aims to serve as a catalyst for further policy discussion and action.

## Conclusion

The seven widely recognized drivers of America’s high health care costs—absence of price limits, reliance on fee-for-service models, inflated specialist salaries, excessive administrative expenditures, opaque pricing structures, high drug prices, and the influence of private equity—remain critical challenges requiring policy attention. However, concentrating exclusively on these visible issues, while neglecting the deeper systemic causes on both the supply and demand sides of the national health care system, risks repeating past reform failures.

Historical experience underscores that effective national reform requires a holistic approach that addresses both the observable symptoms of fragmentation on the one hand and the deeper structural forces underlying cost inflation in the US health care system on the other hand. Equally important is the establishment of a cooperative political environment, the absence of which continues to hinder meaningful progress.

In the near term, irrespective of partisan control, targeted and technological driven measures can produce immediate benefits while creating the foundation for broader reform. These include leveraging AI innovations to enhance price transparency, reforming payment models to align incentives with value, and addressing inefficiencies in the health care labor market. Collectively, such steps can promote affordability and sustainability. Ultimately, only by effectively addressing both visible and hidden cost drivers can policymakers design reforms that ensure the US health care system is both high-quality and accessible for all.

## Data Availability

No datasets were generated or analysed during the current study.
